# Circularly Polarized Near‐Infrared Electroluminescence from Chromium(III) Complex‐Based OLEDs

**DOI:** 10.1002/smll.202512638

**Published:** 2025-11-18

**Authors:** Maxime Poncet, Juan‐Ramón Jiménez, Francesco Zinna, Claude Piguet, Lorenzo Di Bari, Chiara Botta, Umberto Giovanella

**Affiliations:** ^1^ Department of Inorganic and Analytical Chemistry University of Geneva 30 quai Ernest Ansermet Geneva 4 CH‐1211 Switzerland; ^2^ Departamento de Química inorgánica, Facultad de Ciencias Universidad de Granada and Unidad de Excelencia en Química (UEQ) Avda. Fuente Nueva s/n Granada 18071 Spain; ^3^ Dipartimento di Chimica e Chimica Industriale Università di Pisa via Moruzzi 13 Pisa 56126 Italy; ^4^ Istituto di Scienze e Tecnologie Chimiche “Giulio Natta” (CNR‐SCITEC) Consiglio Nazionale delle Ricerche via A. Corti 12 Milano 20133 Italy

**Keywords:** chromium, CPL, CP‐NIR‐OLEDs, electroluminescence, spin‐flip

## Abstract

Organic light‐emitting diodes (OLEDs) featuring circularly polarized (CP) electroluminescence (EL) are gaining tremendous attention for their potential in advanced display and photonic applications. Chiral organic molecules and 4d and 5d metal complexes have been extensively studied for their role in the emissive layer of these devices. Here, pioneering work demonstrating EL emission from a chromium(III) complex is presented. Notably, by leveraging the highly polarized spin‐flip transitions of enantiopure Cr^III^ complexes, a proof‐of‐concept device showcasing near‐infrared (NIR) CP EL with peaks at 726 and 747 nm and corresponding dissymmetry values up to 0.015 and −0.029 is developed. These findings highlight the potential of earth‐abundant Cr^III^ complexes for cutting‐edge chiral optoelectronic applications in medicine, security, and quantum communications.

## Graphical abstract

Electroluminescence is developed from a chromium(III) complex, showing near‐infrared circularly polarized emission at 726 and 747 nm. This highlights the promise of earth‐abundant chromium(III) complexes for advanced chiral optoelectronic applications in fields like medicine, security, and quantum communications.

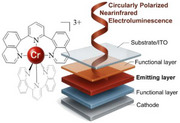

## 1 Introduction

**Figure 1:**
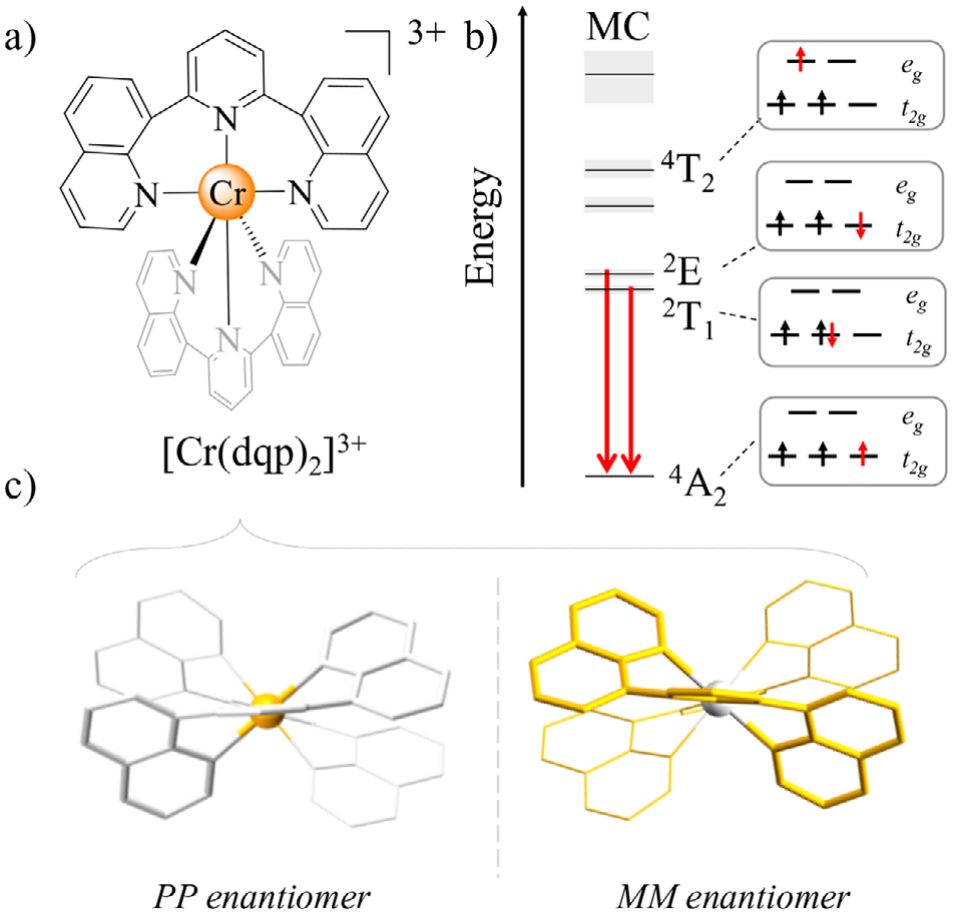
a) Molecular structure of [Cr(dqp)_2_]^3+^ complex; b) Simplified MC energy diagram highlighting the SF transitions. c) View of the two enantiomers present in the racemic mixture.

CP EL from OLEDs has garnered significant interest due to its potential applications in next‐generation display and photonic technologies. CP‐OLED can reduce energy losses in displays, where anti‐glare layers based on CP filters reduce the transmission of light from conventional unpolarized LEDs.^[^ ^1^, ^2^ ^]^ Chiral organic molecules and 4d and 5d‐based metal complexes were extensively investigated as components of the emissive layer in these devices.^[^ ^2^, ^3^, ^4^, ^5^ ^]^ However, they usually suffer from low photoluminescence (PL) and PL dissymmetry factors (*g*_PL_, which quantifies the degree of handedness of CP emission, see Equation (1), because of the relatively strong electric dipole character of their radiative transitions.^[^ ^6^ ^]^ Alternatively, chiral lanthanide complexes exploiting electric‐dipole forbidden but magnetic‐dipole allowed *f–f* transition generating highly dissymmetric CP PL (CP‐PL) (up to g_PL_ ‐1.54 in solution for Eu^III^ based systems) are the most promising examples.^[^ ^6^, ^7^, ^8^ ^]^ Research on less critical chiral materials is advancing rapidly, driven by the need for environmentally friendly alternatives in photophysical and photochemical applications. In this context, Zn^II^‐ and Cu^I^‐based complexes have gained attention as promising candidates due to their abundance in the Earth's crust and luminescence. However, while these materials can exhibit efficient ligand‐centered (LC) and metal‐to‐ligand charge transfer (MLCT) emission, they often suffer from low *g*_PL_ which limits their effectiveness in chiroptical applications.^[^ ^9^, ^10^, ^11^, ^12^, ^13^, ^14^, ^15^ ^]^ Alternatively, Cr^III^‐based materials provide a compelling approach by leveraging spin‐flip (SF) transitions, which exhibit a *g*_PL_ up to two orders of magnitude higher than their copper and zinc counterparts.^[^ ^16^, ^17^ ^]^ Cr^III^ has played a crucial role in doped solids, such as Cr^III^:α‐Al_2_O_3_ (ruby), where its strong phosphorescence at 694.3 nm enabled the development of the first pulsed laser.^[^ ^18^ ^]^ Since then, a large library of Cr^III^‐doped materials with persistent NIR luminescence has been reported.^[^ ^19^, ^20^, ^21^, ^22^ ^]^ The characteristic metal‐centered (MC) emission of Cr^III^ (**Figure** **1**b) commonly occurs within the 680–800 nm wavelength range. These SF transitions are both orbital‐ and spin‐forbidden, leading to a significantly extended emission lifetime due to their lower radiative transition probabilities. Furthermore, the electric‐dipole‐forbidden nature of this transition brings its magnitude comparable with that of the magnetic‐dipole transition moment, a crucial factor in enhancing *g*_PL_.^[^ ^23^ ^]^ The transition from Cr^III^‐doped solids to molecular metal complexes improves solubility and processability, enabling easier integration into device architectures. However, this shift also introduces competing non‐radiative pathways, including molecular vibrations and solvent interactions, which can substantially reduce luminescence efficiency.^[^ ^24^ ^]^ Optimizing ligand design is therefore crucial, and the use of six‐membered terdentate ligands has proven to be a successful strategy for improving the photophysical properties of Cr^III^ complexes in both organic and aqueous solutions.^[^ ^25^ ^]^ These novel Cr^III^ molecular compounds have been exploited for various applications.^[^ ^26^, ^27^, ^28^, ^29^, ^30^, ^31^ ^]^ In 2019, new possibilities emerged with the successful chiral resolution of the racemic [Cr(dqp)_2_]^3+^ complex (dqp = 2,6‐di(quinolin‐8‐yl)pyridine), hereafter *(Rac)*‐[Cr(dqp)_2_]^3+^ (Figure 1a–c). This advancement led to dual CP emission with opposite polarizations at the edge of the NIR region, corresponding to the SF Cr(^2^E(1)→^4^A_2_) and Cr(^2^T_1_(1)→^4^A_2_) transitions.^[^ ^32^, ^33^, ^34^, ^35^ ^]^

The observed g_PL_ values of 0.1 and 0.2, accompanied by a relatively high PL quantum yield (PLQY) of up to 17%, highlight the potential of chiral Cr^III^ emitters in this field.^[^ ^16^, ^32^, ^33^, ^34^, ^35^, ^36^ ^]^ Moreover, Cr^III^ compounds offer significant advantages: i) they serve as a cost‐effective alternative to precious metals (4d and 5d) as dopant agents in the chiral layer, and ii) their kinetic inertness guarantees stability and adaptability across diverse chemical environments, a characteristic that is sometimes compromised in more labile 4f‐based complexes.^[^ ^37^ ^]^

NIR‐emitting CP‐OLEDs (CP‐NIR‐OLEDs)^[^ ^38^ ^]^ are attracting interest for bio‐imaging and bio‐assays in the 650–1350 nm range, but reports are limited, mostly involving Pt‐complexes^[^ ^39^ ^]^ or extended conjugated organics.^[^ ^38^, ^40^ ^]^

This work reports the first EL from a Cr^III^ complex (Figure 1a) and demonstrates OLEDs that emit CP‐NIR light when enantiopure Cr^III^ complexes (Figure 1c) are used as emitters. The devices we present serve as a proof‐of‐concept of the feasibility of CP‐OLEDs based on Cr^III^ SP transitions, with CP‐EL comparable to, or even surpassing, those achieved with other, more established chiral deep red/NIR emitting transition metal complexes.^[^ ^14^ ^]^ On the other hand, this work is not primarily focused on achieving competitive external quantum efficiencies (EQE), which will require further optimization.

## 2 Results

[Cr(dqp)_2_]^3+^ complex was chosen for its suitable photophysical and chiroptical properties, as reported by some of us,^[^ ^35^ ^]^ associated with its two main SF transitions (Figure 1b) in the 700–800 nm spectral region. The two tridentate dqp ligands form a helix‐like structure around the Cr center, defining an overall *D_2_*‐symmetry (Figure 1c). *D_2_*‐symmetry is particularly beneficial for chiroptical properties, as it requires that electric (**µ**) and magnetic (**m**) transition dipoles be always (anti‐)parallel (θ_**µm**_ = 0°,  180°), thus maximizing the dissymmetry factor g_PL_ (Equation (1)):^[^ ^33^, ^34^ ^]^

(1) $$g_{\mathrm{PL}}=4\frac{\left|\mu \right|\cdot \left|m\right|}{\left({\left|\mu \right|}^{2}+{\left|m\right|}^{2}\right)}\cos\theta_{\mu m}$$

The racemic mixture *(Rac)*‐[Cr(dqp)_2_](PF_6_)_3_ was easily prepared and optically resolved using a chiral stationary phase HPLC following the previously reported protocol.^[^ ^35^, ^41^ ^]^ The two enantiomers *(MM)*‐[Cr(dqp)_2_](PF_6_)_3_ and *(PP)*‐[Cr(dqp)_2_](PF_6_)_3_ (hereafter *MM* and *PP*, respectively) were isolated with high optical purity.

*(Rac)*‐[Cr(dqp)_2_](PF_6_)_3_ is initially employed to demonstrate EL and to validate device architectures for enantiomer‐based devices. In view of the assessment in OLED applications, *(Rac)*‐[Cr(dqp)_2_](PF_6_)_3,_ and the enantiopure *MM* and *PP* complexes are preferentially processed via a host‐guest approach to produce thin films, identifying a suitable host matrix in which they can be dispersed. The host comprises a commercially available 4,4′‐Bis(N‐carbazolyl)‐1,1′‐biphenyl (CBP) and 1,3‐Bis[2‐(4‐tert‐butylphenyl)‐1,3,4‐oxadiazo‐5‐yl]benzene (OXD7) mixture,^[^ ^42^ ^]^ which can provide appropriate bipolar charge transport to promote emission centered on the Cr^III^‐complex.

To determine the optimal conditions for Cr^III^ complex‐based OLED fabrication, including active layer formulation and device architecture, we started from the racemic blend CBP:OXD7:*(Rac)*‐[Cr(dqp)_2_]^3+^ in a mass ratio (1:1:0.2), deposited from a degassed acetonitrile:dichloromethane (1:5) mixed solution to form a thin (≈50 nm) film.

**Figure 2:**
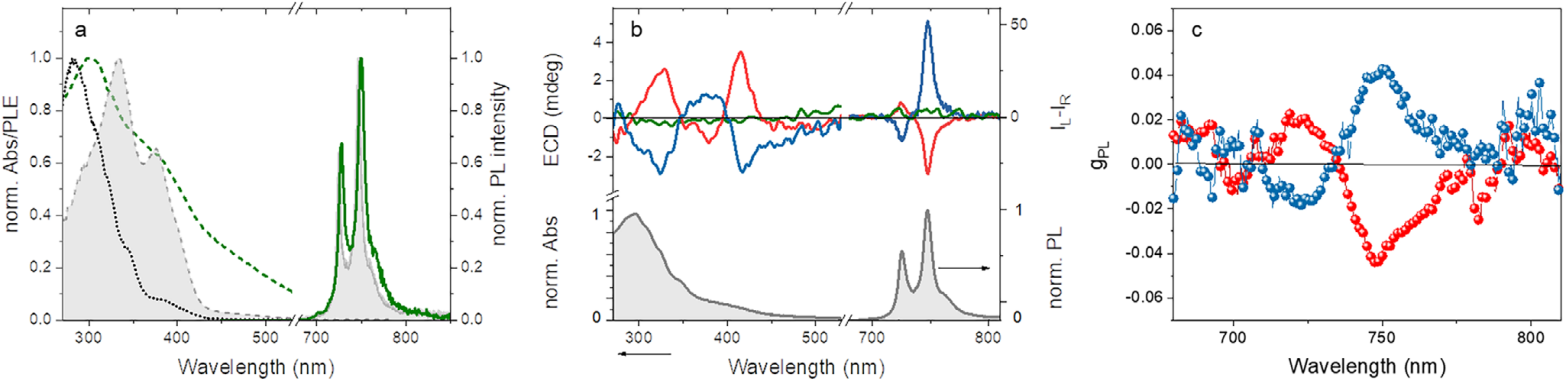
Photophysics and chiroptical properties. a) Absorption (shadowed grey dashed line) and PL (grey solid line) of *(Rac)*‐[Cr(dqp)_2_]^3+^ acetonitrile solution together with absorption (dashed green line), phosphorescence spectrum (solid green line, measured by exciting at 290 nm, with delays of 200 µs, integration of 1 ms), and PLE (black dotted line, monitored at 747 nm) of CDB:OXD7:*(Rac)*‐[Cr(dqp)_2_]^3+^ film; b) ECD and CP‐PL of films of *MM* (blue lines) and *PP* enantiomers (red lines), compared with *(Rac)*‐[Cr(dqp)_2_]^3+^ (green lines) blended with CDB:OXD7, together with representative Abs and PL spectra of *PP* blend film (bottom); c) g_PL_ of *MM* (blue lines and symbols) and *PP* (red lines and symbols) enantiomers blend films.

*(Rac)*‐[Cr(dqp)_2_]^3+^ blend film absorption is dominated by the host matrix features (main peak at 282 nm, Figure S1a, Supporting Information), while the Cr^III^ complex contribution appears as a weak band in the range of 350–420 nm (**Figure** **2**a) with no notable chiroptical activity (Figure 2b). Once excited at 300 nm, the film shows PL emission (Figure S1b, Supporting Information) from both host matrix (band at 410 nm) and Cr^III^ complex, with peaks at 726 and 747 nm corresponding to SF Cr(^2^E→^4^A_2_) and Cr(^2^T_1_→^4^A_2_) transitions, respectively.^[^ ^43^ ^]^ These two peaks are also present in the phosphorescence emission spectrum measured under 290 nm excitation and acquired with delays of 200 µs (Figure 2a). The PL excitation spectra (PLE) recorded by monitoring emission at 726 and 747 nm peaks are identical and closely match the corresponding absorption. This indicates that resonant energy transfer from the host to the Cr^III^ complex, beneficial for device performance, takes place. However, a minor reduction of PL emission (PLQY = 0.5%) and shortening of PL decay time (average lifetimes of 0.45 ms, from multiexponential fits, Figure S2, Supporting Information) in ambient conditions is observed in Cr^III^‐centered dual phosphorescence within the blend compared to the solution.^[^ ^35^ ^]^ This behavior is consistent with the presence of microaggregates in the blend; in fact, we observed a slight increase in average lifetimes when the matrix was excited, with emission occurring from the well‐dispersed complexes following energy transfer (Figure S2 and Table S1, Supporting Information).

The optical behavior of enantiopure Cr^III^ complexes is very similar to *(Rac)*‐[Cr(dqp)_2_]^3+^ under identical blend formulation (Figure 2b; Figure S3, Supporting Information), but additionally demonstrates chiroptical signals. Both the electronic circular dichroism (ECD) and CP‐PL spectra of the *PP* and *MM* complexes, examined as neat (Figure S4, Supporting Information) and in blend films, display mirror image bands (Figure 2b). The ECD spectra of the blend films are very similar to the ones previously reported for acetonitrile solutions, exhibiting well‐resolved transitions in the 250–500 nm attributed to LC (*π*→π_*_), LMCT, and MC transitions (Figure 2b).^[^ ^35^ ^]^ Upon unpolarized excitation at 360 nm, *PP* and *MM* blend films exhibit two CP emission bands at 726 and 747 nm (Figure 2b). The PL dissymmetry factor g_PL_, yielded by the SF transitions in [Cr(dqp)_2_]^3+^, is positive for the 726 nm band and negative for the 747 nm band in the case of the *PP* enantiomer (Figure 2c). Conversely, the signs are opposite for the *MM* enantiomer. The maximum values of |g_PL_| for these transitions are 0.020 and 0.043, respectively (**Table** **1**).

**Table 1:** Summary of maximum dissymmetry values of blend films and devices at 726/747 nm.

CBP:OXD7 blend	g_PL_	g_EL_
*PP* enantiomer	0.020/−0.043	0.015/−0.029
*MM* enantiomer	−0.018 / 0.043	−0.015 / 0.010

Although there have been no prior reports of electroluminescent Cr^III^ compounds, the distinctive chiroptical properties, despite the moderate PLQY, displayed by the *MM* and *PP* complexes indicate that these materials could be promising candidates for evaluation in CP‐OLEDs.

Prototypes are fabricated by following a multilayered approach^[^ ^38^, ^44^ ^]^ in a direct bottom‐emitting architecture. Sequential deposition of layers is performed using both solution methods and vacuum growth. Electrodes are engineered with functional layers of semiconducting compounds that mitigate energy barriers for charge injection, facilitate charge transport, and confine excitons' radiative recombination within the active layer.^[^ ^44^ ^]^

In Cr^III^ complexes, the concept of a highest occupied molecular orbital (HOMO)–lowest unoccupied molecular orbital (LUMO) gap requires careful interpretation due to the open‐shell d^3^ electronic configuration. In a simplified picture, the highest occupied orbitals correspond to the singly occupied t_2g_ orbitals (SOMOs), while the lowest unoccupied orbitals are the empty e_g_^∗^ orbitals. The energy difference between these orbitals reflects the ligand‐field splitting parameter (Δ_0_) in octahedral symmetry (Figure 1b). It can be assessed by the metal‐centered Cr(^4^T_2_←^4^A_2_) transition typically observed in the electronic absorption spectra. For [Cr(dqp)_2_]^3^⁺, this transition appears as a manifold around 420–430 nm (≈3.0 eV),^[^ ^35^, ^41^ ^]^ providing a spectroscopic estimate of the SOMO–LUMO gap. Alternatively, cyclic voltammetry of (*Rac*)‐[Cr(dqp)_2_]^3^⁺ was used to estimate its frontier orbital energies (Figure S5a, Supporting Information). The LUMO level, derived from the first reduction wave, is located at −5.35 eV (vs vacuum). The half‐filled metal‐centered t_2g_ SOMO could not be accessed electrochemically (no oxidation wave was observed within the solvent window); instead, its energy was inferred from the optical band gap, yielding an approximate value of −2.11 eV. Both approaches converge on a gap in the range of 3.0 eV (Figure S5b, Supporting Information).

**Figure 3:**
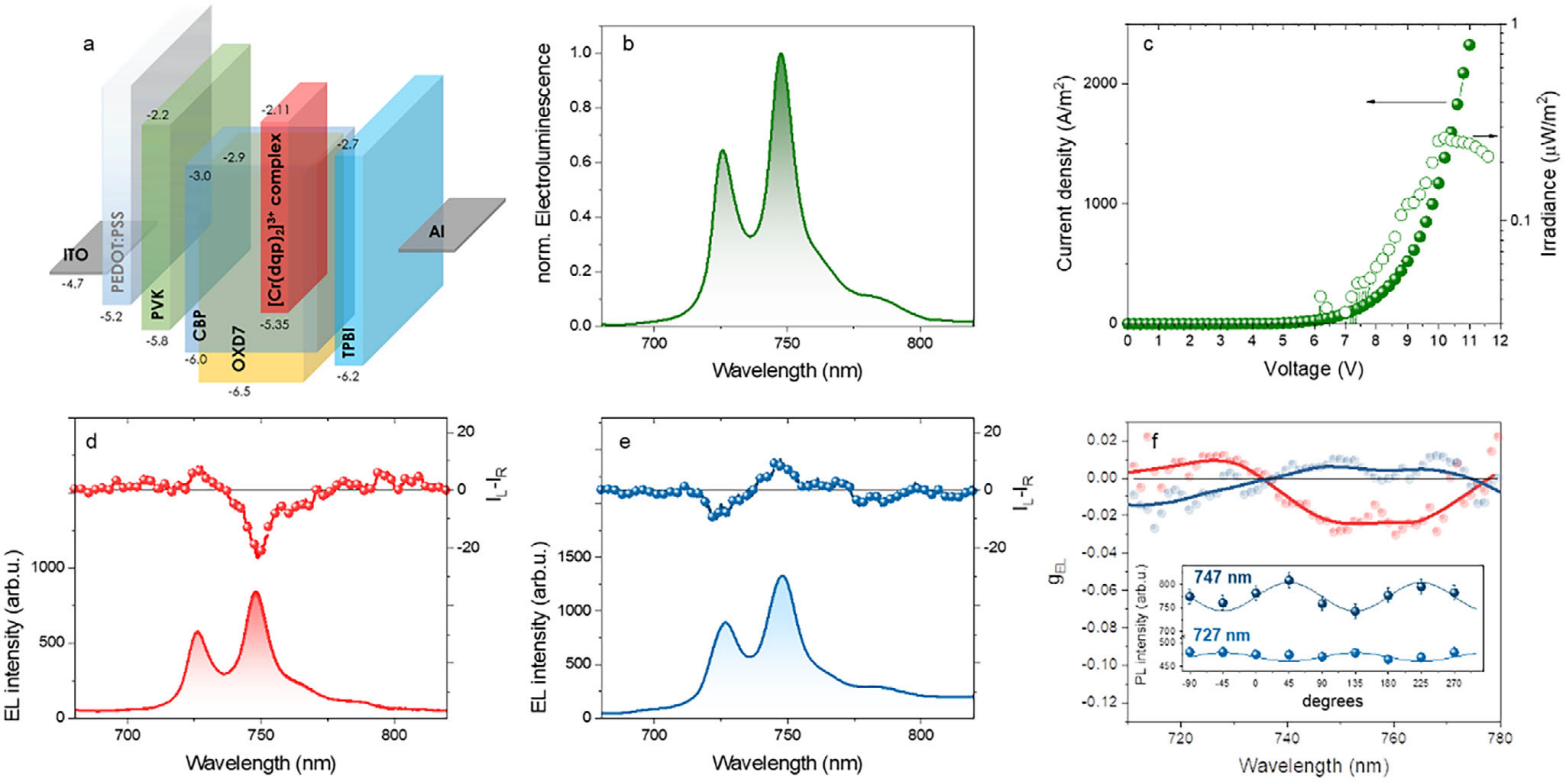
CP‐NIR‐OLEDs. a) flat‐band energy level diagram of CP‐NIR‐OLED components; b) EL spectrum of ITO/PEDOT:PSS/PVK/CBP:OXD7:*(Rac)*‐[Cr(dqp)_2_]^3+^complex/LiF/Al; c) current density/irradiance/voltage characteristic curves; EL and CP‐EL of d) *PP* enantiomer and e) *MM* enantiomer‐based CP‐NIR‐OLEDs and f) corresponding g_EL_ vs wavelength plot; inset, oscillation pattern of *MM* complex‐based CP‐NIR‐OLED as a function of the angle *θ* of the easy axis of the rotating QWP with respect to the axis of the fixed LP.

The OLED architecture ITO/PEDOT:PSS/PVK/AL/TPBI/LiF/Al is initially tested with *(Rac)*‐[Cr(dqp)_2_]^3+^ as the emitter dispersed in CBP:OXD7 host. The flat‐band energy diagram of all components is reported in **Figure** **3**a. Upon direct biasing, the OLEDs exhibit EL with the two characteristic peaks at ≈727 and 747 nm from Cr^III^‐centered emission (Figure 3b). The devices switch on at ≈6 V with an external quantum efficiency of ≈10^−3^% and a maximum irradiance of 0.29 µW m^−2^ at 10 V (Figure 3c).

To the best of our knowledge, this is the first report of EL from a Cr^III^ complex. Notably, *(Rac)*‐[Cr(dqp)_2_]^3+^ can produce EL emission also as a neat active layer film (Figure S6, Supporting Information), but the host‐guest approach ensures higher performance (Figure 3c; Figure S7, Supporting Information) and longer operational stability over the measurement procedures.

When *MM* and *PP* complexes are used as emitters, following the same device manufacturing protocol, CP‐EL is observed (Figure 3d,e). To prevent polarization cancellation due to reflection on the back (cathode) electrode,^[^ ^44^, ^45^ ^]^ a semitransparent Al cathode (thickness ≈8 nm) is employed. Although this can limit device efficiency and slightly increase the turn‐on voltage,^[^ ^46^ ^]^ g_EL_ values of 0.015 and −0.029 at 726 and 747 nm, respectively (Figure 3f), are obtained for a *PP*‐based CP‐NIR‐OLED. Similar, but mirror image, CP‐EL spectra are obtained for devices containing the *MM* enantiomer (Figure 3f and Table 1). Notably, the dissymmetry peaks are located at the edge of the NIR window, with g_EL_ values surpassing those observed for reported chiral emitters in a similar region, such as chiral Pt(II)^[^ ^47^ ^]^ and Cu(I) complexes.^[^ ^48^ ^]^

To further exclude the presence of linearly polarized components in the CP‐EL signal, we measured EL intensity of the two peaks (at 726 and 747 nm) for the *MM*‐based CP‐NIR‐OLEDs as a function of the angle *θ* between the easy axis of the rotating quarter waveplate (QWP) and the fixed linear polarizer (LP). Consistent with expectations, the EL intensity exhibits a periodic variation with a period of 180°, characterized by a cosine/sine‐squared dependence (Equation (2), inset of Figure 3f):

(2) $$I=I_{L}\mathrm{co}s^{2}\left(\theta +45^{\circ }\right)+I_{R}\mathrm{si}n^{2}\left(\theta +45^{\circ }\right)$$

with *θ* in degrees.

Although dual, oppositely‐signed, CP‐EL bands are undesirable in displays, they provide an advantage in CP‐based biosensing, enabling built‐in ratiometric detection and wavelength multiplexing. Moreover, this feature is advantageous for chromaticity‐based CPL detection (CPL photoscopy), where the circular polarization information is encoded directly into colour differences arising from the two oppositely polarized bands.^[^ ^49^ ^]^

## 3 Conclusion

In conclusion, we reported, for the first time, that EL emission from a chromium(III) complex is possible. Moreover, the significant breakthrough lies in the ability to produce polarized photons emitted by the devices. Specifically, we exploited the highly polarized spin‐flip transitions of enantiopure Cr^III^ complexes to fabricate devices displaying NIR CP‐EL. This was accomplished by adhering to a multilayered device manufacturing protocol with engineered electrodes and a proper formulation of the active layer composition. This unprecedented achievement demonstrates the potential of earth‐abundant Cr^III^ complexes not only as a viable alternative to lanthanide‐based CP‐OLEDs but also in advanced chiral optoelectronic applications, quantum computing, and photonic devices.

## 4 Experimental Section

### Materials

The *(Rac)*‐[Cr(dqp)_2_](PF_6_)_3_ was prepared as previously reported.^[^ ^35^, ^41^ ^]^ Enantiopure materials were obtained by chiral stationary phase HPLC resolution on a Shimadzu apparatus (double binary pump, auto sampler, column thermostat, and diode array detector, collection apparatus) using a semi‐preparative CHIRALPAK IC column (250 × 10 mm, 5 µm) and HPLC grade solvents.

### Characterization

Cyclic voltammogram of [Cr(dqp)_2_]^3+^ was recorded in CH_3_CN + 0.1 M NBu_4_PF_6_ (*c* = 10^−3 ^M). Working electrode: mercury; counter Electrode: silver; Reference Electrode: Silver Chloride. Scan rate 100 mV s^−1^. Electronic absorption spectrum was carried out on a Perkin‐Elmer Lambda 900 UV—VIS–NIR Spectrometer. ECD spectra were recorded with a Jasco J‐1500 spectropolarimeter. PL and EL spectra are obtained with a NanoLog composed of an iH320 spectrograph equipped with a Synapse QExtra charge‐coupled device. Excitation is performed with a monochromated 450 W Xe lamp. The spectra are corrected for the instrument response. PLQY of solid‐state samples was measured with a home‐made integrating sphere according to the procedure reported elsewhere.^[^ ^50^ ^]^ Time‐resolved measurements are obtained with the PPD‐850 single photon detector module and analyzed with the instrument Software by using three‐exponential best fits. CP‐EL spectra are obtained by rotating a quarter waveplate inserted between the device and a fixed linear polarizer.

### Devices Fabrication

For the manufacturing of the devices, indium tin oxide (ITO) was used as the anode. After sequential cleaning of 25 mm × 25 mm sized ITO‐coated glasses in acetone and isopropanol in a sonicator, they were treated with a nitrogen plasma. Poly(3,4‐ethylenedioxythiophene) polystyrene sulfonate (PEDOT:PSS) AI 4083 (Ossila) was spin‐coated to form a 35 nm thick film. Polyvinylcarbazole (PVK, Merck KGaA) film was deposited on top of PEDOT:PSS from a 10 mg mL^−1^ anhydrous chlorobenzene solution to form ≈35 nm thick film. Solutions of the 4,4′‐Bis(N‐carbazolyl)‐1,1′‐biphenyl (CBP): 1,3‐Bis[2‐(4‐tert‐butylphenyl)‐1,3,4‐oxadiazo‐5‐yl]benzene (OXD7):Cr^III^ complex blends in acetonitrile:dichloromethane (1:5) at a total concentration of 15 mg mL^−1^ were spin‐coated from a degassed solution (under inert atmosphere) on top of the glass/ITO/PEDOT:PSS/PVK substrate. The 2,2′,2′'‐(1,3,5‐Benzinetriyl)‐tris(1‐phenyl‐1‐H‐benzimidazole) (TPBI, Ossila) electron transport/hole blocking layer (30 nm) was deposited using an Organic Molecular Beam Deposition system. Finally, 1.5 nm of LiF and 8–100 nm of Al were thermo‐sublimated inside the high vacuum evaporator to achieve a semitransparent or mirror cathode. Photons emitted in the forward direction through the glass substrate were collected by a calibrated photodiode. Current density‐light‐voltage curves were recorded by dual‐channels Keithley 2602 apparatus controlled by a home‐made software in a Nitrogen atmosphere without device encapsulation. The radiance of the devices was estimated using the same setup, and Lambertian emission was assumed.

### Statistical Analysis

12 independent devices for each complex were built to check for reproducibility. CP‐EL spectra were acquired by recording at least 8 spectra for each QWP position. The spectra acquired with QWP positions corresponding to left/right CP light were averaged obtaining I_L_/I_R_. CP‐EL was calculated as I_L_‐I_R_, total EL as I_L_‐I_R_, g_EL_ as 2(I_L_‐I_R_)/(I_L_+I_R_). g_EL_ values in the inset of Figure 3f are reported with a relative standard deviation of ≈10%.

## Conflict of Interest

The authors declare no conflict of interest.

## Supporting information



Supporting Information

## Data Availability

The data that support the findings of this study are available in the supplementary material of this article.
