# Supplementary material for: Circularly Polarized Near‐Infrared Electroluminescence from Chromium(III) Complex‐Based OLEDs
Source: Small. 2025 Nov 18;22(1):e12638. doi: 10.1002/smll.202512638 (PMC12757983; doi:10.1002/smll.202512638)
Supplement: Supplementary file 1 — Supporting Information [file SMLL-22-e12638-s001.docx]

Supporting Information

Circularly Polarized Near-infrared Electroluminescence from Chromium(III) Complex-Based OLEDs

Maxime Poncet, Juan-Ramón Jiménez,* Francesco Zinna,* Claude Piguet, Lorenzo Di Bari, Chiara Botta, and Umberto Giovanella*

**Figure S1.** Absorption (Abs, red solid line) and PL excitation spectra (PLE, green dotted line) together with PL emission (by exciting at 300 nm) (solid blue line) of undoped CBP:OXD7 host matrix (a); PL spectrum (by exciting at 300 nm) of CBP:OXD7:*(Rac)*-[Cr(dqp)_2_]^3+^ film (b).

(a) (b)

**Figure S2.** PL decay time from 3-exponential fits, in ambient conditions of Cr^III^-centered dual phosphorescence within the blend. (a) excitation at 290 nm, (b) direct excitation of the complex at 410 nm. From 3-exponential fits, average lifetimes obtained as τ_av_ =$\sum_{i} \frac{A_{i}\tau_{i}^{2}}{A_{i}\tau_{i}}$

ex290 em726, τ_av_ = 586 µs

ex290 em747, τ_av_ = 564 µs

ex410 em726, τ_av_ = 450 µs

ex410 em747, τ_av_ = 452 µs

**Table S1**. Photophysical properties of *(Rac)*-[Cr(dqp)_2_]^3+^ complex solution and blend film.

|  | PLQY  (%) | τ_obs_  (ms) | τ_rad_  (ms) |
| --- | --- | --- | --- |
| *(Rac)*-[Cr(dqp)_2_]^3+^ aqueous solution | 5.2 (deaerated)^[1]^  0.5 (aerated)^[2]^ | 1.2 (deaerated)^[1]^  0.089 (aerated)^[2]^ | 23  18 |
| CBP:OXD7:*(Rac)*-[Cr(dqp)_2_]^3+^ film | 0.5^a^  6.4^b^ | 0.45^c^  0.59^d^ | 90  9 |

^a^ Cr^III^ complex emission; ^b^ total emission of the blend; ^c^ complex direct excitation (410 nm); ^d^ blend excitation (290 nm); Average lifetimes obtained from 3-expoinential fits: estimated relative uncertainty ±10%. PLQY obtained by exciting at 340 nm, estimated relative uncertainty ± 10%. τ_rad_ = τ_obs_ /PLQY

**Figure S3.** Optical properties of *PP* enantiomer (in the same blend formulation as the racemic complex). Absorption (dashed line) and PL (solid line) of *PP* enantiomer in acetonitrile solution (grey lines) and in CBP:OXD7 blend film (red lines).

**Figure S4.** (a,b) ECD spectra and (c,d) g_PL_ dissymmetry factors of the *PP* (a,c) and *MM* enantiomers (b,d), as neat films. PL spectra of the enantiomers are reported (c,d).

**Figure S5.** (a) Cyclic voltammogram of 1 mM [Cr(dqp)_2_]^3+^ in Nitrogen-saturated CH_3_CN against Fc/Fc^+^. 0.1 M TBAPF_6_ was used as supporting electrolyte. A glassy carbon disk was used as a working electrode, and a platinum wire served as the counter electrode. (b) Electronic absorption spectrum of the *(Rac)*-[Cr(dqp)_2_](PF6)^3^ from which the optical band-gap has been estimated.

**Figure S6.** Normalized EL emission (at 10 V) of OLED based on *(Rac)*-[Cr(dqp)2]^3+^ complex as neat active layer.

**Figure S7.** (a) Representative current density/light/voltage characteristic curves and (b) EQE versus current density of a device featuring *(Rac)*-[Cr(dqp)_2_]^3+^ complex as neat active layer.
